# Partial reduction of microglia does not affect tau pathology in aged mice

**DOI:** 10.1186/s12974-018-1348-5

**Published:** 2018-11-09

**Authors:** Rachel E. Bennett, Annie Bryant, Miwei Hu, Ashley B. Robbins, Sarah C. Hopp, Bradley T. Hyman

**Affiliations:** 1 Department of Neurology, MassGeneral Institute for Neurodegenerative Disease, Massachusetts General Hospital, Harvard Medical School, Charlestown, MA 02129 USA; 2 Biggs Institute for Alzheimer’s and Neurodegenerative Disease, University of Texas Health Science Center San Antonio, 7703 Floyd Curl Drive, San Antonio, TX 78229 USA; 3 Department of Pharmacology, University of Texas Health Science Center San Antonio, San Antonio, TX 78229 USA

**Keywords:** Microglia, Alzheimer’s disease, Tau

## Abstract

**Background:**

Activation of inflammation pathways in the brain occurs in Alzheimer’s disease and may contribute to the accumulation and spread of pathological proteins including tau. The goal of this study was to identify how changes in microglia, a key inflammatory cell type, may contribute to tau protein accumulation and pathology-associated changes in immune and non-immune cell processes such as neuronal degeneration, astrocyte physiology, cytokine expression, and blood vessel morphology.

**Methods:**

We used PLX3397 (290 mg/kg), a colony-stimulating factor receptor 1 (CSF1R) inhibitor, to reduce the number of microglia in the brains of a tau-overexpressing mouse model. Mice were fed PLX3397 in chow or a control diet for 3 months beginning at 12 months of age and then were subsequently analyzed for changes in blood vessel morphology by in vivo two-photon microscopy and tissues were collected for biochemistry and histology.

**Results:**

PLX3397 reduced microglial numbers by 30% regardless of genotype compared to control diet-treated mice. No change in tau burden, cortical atrophy, blood vessels, or astrocyte activation was detected. All Tg4510 mice were observed to have an increased in “disease-associated” microglial gene expression, but PLX3397 treatment did not reduce expression of these genes. Surprisingly, PLX3397 treatment resulted in upregulation of *CD68* and *Tgf1β*.

**Conclusions:**

Manipulating microglial activity may not be an effective strategy to combat tau pathological lesions. Higher doses of PLX3397 may be required or earlier intervention in the disease course. Overall, this indicates a need for a better understanding of specific microglial changes and their relation to the disease process.

## Introduction

An increase in the number of reactive microglia is a key feature of Alzheimer’s disease (AD, [35]), with data from multiple genome-wide association studies confirming these cells are important to disease pathogenesis [9, 40]. In particular, increased numbers of microglia are frequently found both surrounding amyloid β (Aβ) plaques and in brain regions where neurofibrillary tangles containing tau are present [3, 34, 36]. The role of microglia near these pathological lesions is believed to aid clearance of toxic protein aggregates [27, 28]. On the other hand, microglia may influence neurodegenerative phenotypes including tau spreading and synaptic loss, and microglial secretion of inflammatory factors may exacerbate pathology [5, 11, 19, 22, 24, 32]. Microglia activation induces neuronal tau phosphorylation via p38 MAPK [30], and reduction of microglia activation is capable of reducing neuronal tau phosphorylation in vivo [16]. Taken together, it is unclear how microglia influence AD. Recent studies have identified gene expression changes in microglia from Alzheimer’s disease brain and mouse models indicating that the subset of microglia near plaques are phenotypically distinct from non-plaque-associated microglia—highlighting the importance of understanding the diversity of responses to disease pathology [18, 21].

To better understand if microglia change neurofibrillary tangle pathology, we investigated the relationship between the presence of microglia, tau protein accumulation, and other tau-related pathological events. We have previously observed changes in blood vessel morphology that take place between 12 to 15 months of age and hypothesized that microglia-mediated inflammatory pathways may play a role at this time point [4]. Microglia are capable of phagocytosing tau aggregates and have been hypothesized to contribute to the pathological spread of tau in the brain by inefficient degradation of tau seeds [14]. Bhaskar et al. found that mice lacking a microglial CX3CR1 fractalkine receptor had enhanced tau phosphorylation via increased activity of p38 MAPK [5, 30]. Separately, others have used clodronate liposomes or the colony-stimulating factor 1 receptor (CSF1R) inhibitors PLX3397 or PLX5622 to deplete microglia from the brains of mouse models of AD. In one study of early tau accumulation, depletion of microglia in mice expressing tau in entorhinal cortex reduced pathological tau spread along axons to the hippocampal dentate gyrus as well as AT8 tau phosphorylation in young PS19 mice [1]. In a separate model, in aged 3xTg mice which develop both Aβ plaques and tangles, microglial depletion appeared to have a minimal effect on measures of Aβ plaque pathology and no effect on tau pathology assessed by either total tau or AT8 phosphorylation [8]. In another AD mouse model, such as the 5XFAD line which only develops plaque pathology, early microglia depletion reduces pathological accumulation of Aβ [37] but later depletion does not, and only has a modest protective effect on neurons [38].

In light of these observations of the complex role of microglia in AD mouse models, we sought to address two questions. First, we asked if tau pathology induces “disease-associated” microglia gene expression changes similar to those reported for microglia in Aβ plaque depositing mice [18, 21]. This was of particular interest due to the apparent resistance of plaque-associated microglia to elimination by CSF1R inhibitors: in one study, PLX3397 depleted 80–90% of total microglia throughout the brain, but only 50% of plaque-associated microglia [38]. Second, we hypothesized that depletion of microglia with PLX3397 in aged mice with tau pathology may reduce pathological features of tau accumulation, particularly in light of the role of microglia in tau phosphorylation, tau accumulation, astrocyte activation, and blood vessel pathology [4], which are all features of the Tg4510 mouse model.

## Methods

### Animals

All mice used in these studies were housed and maintained under the McLaughlin Research Institute or Massachusetts General Hospital Animal Use & Care Committee. In total, 33 Tg4510 (129S6.Cg-Tg(Camk2a-tTA)1Mmay/JlwsJ; FVB-Tg(tetO-MAPT*P301L)Kha/JlwsJ) and litter-mate controls (129S6-Tg(Camk2a-tTA)1Mmay/JlwsJ) were used in these studies and were originally obtained from Jackson Laboratory (Bar Harbor, ME) [33]. PLX3397 was synthesized by Plexxikon and custom blended with a commercially available formulation enriched for vitamins (TK-97184, Teklad). Mice were randomly assigned PLX3397 (PLX; 290 mg/kg) diet or control diet at 12 months of age and each group was balanced for gender. Mice were maintained on each diet for 3 months.

### Histology

Prior to slicing, fixed hemispheres were equilibrated in 30% sucrose in PBS for 24 h. Then, hemispheres were frozen and coronally sliced on a freezing microtome. Ten sets of 40-μm-thick sections were collected such that each set contained sections at 400 μm intervals. Sections were stored in 30% glycerol in PBS at – 20 °C until use. For Iba-1 immunohistochemistry, sections were rinsed in Tris-buffered saline (TBS), incubated in 0.3% hydrogen peroxide for 10 min, then blocked in 3% normal goat serum (NGS) in 0.25% Triton-x TBS (TBS-X) for 30 min prior to incubation in goat anti-Iba-1 (1:1000, WAKO) overnight at 4 °C in blocking solution. The following day, sections were washed in TBS, incubated in donkey anti-goat IgG in 0.25% TBS-X for 1 h, then washed and incubated with streptavidin-HRP (Vector Labs ABC) for 30 min, and subsequently washed and developed with diaminobenzidine containing NiCl_2_ for 4–5 min. Following staining, sections were washed, mounted on glass slides, and then dehydrated in ascending ethanol/xylene and coverslipped with Cytoseal XYL (Thermo Fisher Scientific). For triple labeling, sections were treated as before then incubated in rabbit anti-NeuN (1:1000, Millipore), mouse anti-tau (AT8, 1:1000, Thermo Fisher Scientific), and chicken anti-GFAP (1:1000, Millipore) in blocking solution overnight at 4 °C. The following day, sections were incubated for 1 h with goat anti-mouse IgG Alexa488, goat anti-rabbit IgG Alexa 555, and goat anti-chicken IgG Alexa 647 (1:2000, Thermo Fisher Scientific) in TBS-X. Sections were then washed and mounted on glass slides and allowed to dry prior to coverslipping with Fluoromount G (Southern Biotech).

### Stereology and image analysis

For stereological quantification of Iba-1 positive microglia, a brightfield microscope fitted with a motorized stage was used along with computer-assisted stereological toolbox version 2.3.1.5 (Olympus America). Slides were first viewed with a × 1.25 objective to identify the anterior-most section in each series containing hippocampus and dentate gyrus was chosen as the first section for analysis and following two posterior sections were also included for a total of three slices. Dorsal cortex was then outlined beginning at midline and extending laterally to the level of the dorsal thalamus. This ROI was chosen as it overlapped best with two-photon measures. In total, 8% of the total area of these regions was counted via stereological random sampling. In a separate set of sections, for stereological analysis of NeuN, 1% of the area was sampled and for AT8, 8% of the area was sampled while the images were viewed by epifluorescence. These sections were also imaged using a VS120 Olympus slide scanner to quantify GFAP (Alexa 647). A threshold-based ImageJ approach was used to quantify the total GFAP + as a percent of the total ROI. Briefly, a DAPI image was used to outline cortex as described above and then the “moments” threshold was applied to all GFAP images. The thresholded GFAP area and total outlined area was then measured per image.

For analysis of blood vessel density and length from two-photon z-stack images, all processing was done in ImageJ as described previously. In short, the 3D Objects Counter was used to measure blood vessel volume and the Skeletonize (2D/3D) plugin was used to measure blood vessel length. All measures were normalized to average cortical thickness (measured from Iba-1 stained sections) to account for atrophy.

### Western blots

To prepare brain protein homogenates, the cerebellum and brain stem was removed and the remaining hemisphere was placed in a 2 ml glass dounce homogenizer with 200 μl PBS containing protease inhibitors (Complete Mini, Roche). Tissue was homogenized with 30 up/down strokes. The homogenate was then centrifuged at 3000×*g* and the supernatant was reserved for analysis. For Western blotting, 10 μg of protein was boiled for 5 min in SDS reducing agent and loading dye (Invitrogen) and then was loaded on a 4% to 12% bis-tris SDS-PAGE in MES buffer (Invitrogen). Samples were run at 120 V for 90 min then transferred to nitrocellulose. Membranes were blocked using Odyssey blocking reagent (Li-Cor) for 30 min then incubated overnight in primary antibodies. Antibodies used in these experiments included mouse anti-tau (AT8, 1:1000, Thermo Fisher Scientific), rabbit anti-phospho tau T231 (1:1000, Invitrogen), chicken anti-Gapdh (1:5000, Millipore), mouse anti-p38 MAPK (1:1000, Cell Signaling Technology), and rabbit anti-phospho-p38 MAPK (1:1000, Cell Signaling Technology). Infrared-labeled secondary antibodies (1:5000, Li-Cor) were then incubated with membranes and detected with a Li-Cor imaging system.

### Cell-based tau seeding activity assay

To assess the presence of bioactive tau “seeds,” we used a fluorescence resonance energy transfer (FRET) cell-based assay [13]. In this assay, HEK293 cells stably express the tau repeat domain (TauRD) conjugated to a yellow fluorescent protein (YFP) or cerulean fluorescent protein (CFP). CFP to YFP FRET at 535 nm can be measured within these cells using flow cytometry after application of tau aggregates or “seeds.” For the experiments herein, cells were plated at 20,000 cells per well in a 96-well plate coated with poly-d-lysine (PDL; 50 μg/ml, Sigma) for at least 3 h. The day after plating, brain lysate from rTg4510 or WT mice was added to the wells at 0.5 μg/well in Opti-MEM medium (#11058-021, Life Technologies) with 1% Lipofectamine 2000 (#11668019, Life Technologies) to promote internalization of tau seeds. Twenty-four hours later, cells were detached with trypsin, transferred to round-bottom plates, centrifuged at 300 g, fixed with 2% paraformaldehyde (PFA) in suspension for 10 min, centrifuged at 300 g, and then finally resuspended in phosphate-buffered saline (PBS). Fixed cells were then analyzed on a MACSQuant VYB (Miltenyi Biotec) flow cytometer with excitation with a 405 nm laser and emission captured by a 525/20 nm band pass filter. Gates were drawn to select live cells by forward and side scatter channels and subsequently single cells (singlets) by forward scatter area and height. Then, 20,000 singlets were collected for analysis. Integrated FRET density (IFD) was calculated by multiplying the median fluorescence intensity of FRET-positive singlets by the percentage of FRET-positive events within the singlets gate. Each flow condition was performed in at least triplicate.

### Quantitative PCR

RNA was obtained using the Qiagen RNeasy Mini Kit (Qiagen Cat. No. 74104). All contact surfaces were cleaned with 70% Ethanol and RNaseZAP (Sigma-Aldrich, Cat. No. R2020). Tissue was homogenized with a mortar and pestle over dry ice and 30 mg was weighed and separated into a 1.5 ml RNase, DNase free Eppendorf tube on ice. Further, 600 μl of lysis buffer (RLT buffer, Qiagen) was added to each tube and the samples was sonicated at 10% amplitude for 30 s. Samples were then spun at 13,000 rpm for 3 min and the supernatant was transferred to a new tube for RNA isolation. On-column RNA isolation was performed according to the RNeasy Mini Kit manufacturer’s instructions and the final RNA sample was eluted in 30 μl of DNase, RNase free water. Final yield was quantified using a Nanodrop spectrophotometer.

All cDNA synthesis reactions were performed with 100 ng of total RNA per sample using the QuantiTect Reverse Transcription Kit (Qiagen Cat. No. 205311). The cDNA synthesis reactions were then added to a 96-well plate with QuantiTect SYBR Green Mastermix (Qiagen Cat. No. 204143), sealed with optically clear flat seal caps and spun at 300×g briefly. All qPCR reactions were performed in a Bio-Rad CFX96 real-time cycler [15 min at 95 °C, 40 cycles (15 s at 94 °C; 30 s at 55 °C; 30 s at 72 °C)]. QuantiTect primer assays were used for the following gene targets: *Actb*, *Gapdh*, *Hprt*, *Icam1*, *Icam2*, *Tjp1*, *Cldn5*, *Ocln1*, *Vcam1*, *Il*-*beta*, *Serpine1*, and *Plau* (Qiagen, Product No. 249900).

A custom TaqMan Fast 96-well qPCR array plate (Applied Biosystems) was designed to examine six microglia genes based on current literature highlighting a specific neurodegenerative phenotype found in microglia in AD and other neurodegenerative mouse models: Cx3cr1, Apoe, Trem2, Il1b, CD68, and Tgfb1. The arrays used Gapdh as a reference gene and 18s rRNA as an internal control. Each plate contained a TaqMan probe and PCR primer set dried down in each well. cDNA was added to each well diluted in 2x TaqMan Fast Universal PCR master mix (Applied Biosystems, 4352042) and ultrapure water. Plates were analyzed on a Bio-Rad CFX96 qPCR machine; Enxymes were activated for 20 s at 95 °C, then 40 cycles of denaturing (95 °C, 3 s) and annealing/extending (30 s, 60 °C) followed by data collection were performed.

### In vivo imaging of blood vessels

At 15 months, acute cranial windows were performed as described previously [4]. Mice were injected with intravenous 70 kDa dextran conjugated to Texas Red to image blood vessels. All two-photon images were acquired over the somatosensory and motor cortex using an Olympus two-photon imaging system equipped with a water immersion lens (25x, N.A. = 1.05) and a Ti:Sapphire (MaiTai, Spectra Physics) tunable laser at 900 nm. For each mouse, six randomly sampled regions of interest (ROIs) were acquired (three per hemisphere). Each ROI was acquired at 3 × digital zoom, beginning 50–100 μm below the surface of the brain to avoid surgery artifacts. A total of 50 slice images were captured at 2 μm steps per ROI. Immediately following imaging, mice were euthanized and perfused with 10 ml cold PBS. One hemisphere was drop-fixed in 4% paraformaldehyde and the other hemisphere was snap frozen and stored at − 80 °C for biochemistry/RNA analysis.

### Statistics

All data were first confirmed to be normally distributed using Shapiro-Wilkes normality test. For ELISA data analyses, a two-tailed student’s *t* test was used. In all other cases, a two-way ANOVA was used to determine overall significance of genotype and treatment followed by post-hoc Sidak’s multiple comparisons. A threshold of *p* < 0.05 was used to determine significance.

## Results

### Reduction of microglia in Tg4510 mice after CSF-1r blockade

**Fig. 1:**
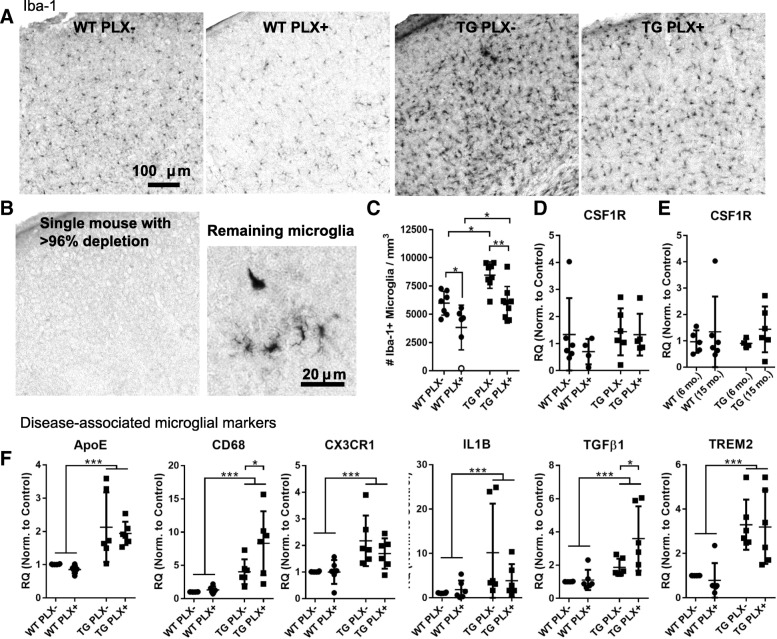
Partial microglial depletion by PLX3397 in aged Tg4510 mice. **a** Representative images of Iba-1 positive microglia in the somatosensory cortex of wild-type (WT) and Tg4510 (TG) mice treated with and without PLX. **b** Iba-1 labeling in a single PLX treated wild-type mouse was near completely absent in the cortex, though deeper layers had occasional ramified and amoeboid cells. **c** The total number of microglia in cortex per mm^3^ were quantified by stereology. The mouse from (**b**) is indicated by an open circle. **d** CSF1R gene expression was measured by qPCR in PLX treated and untreated mice as well as in a separate 6 month (**e**) group of mice. Fifteen month mice in panel (**e**) are the same as those in panel (**d**) and were run on the same qPCR plate. **f** Six different disease-associate microglial markers were assessed by qPCR from PLX treated and untreated mice. All were elevated in Tg4510 animals. All data were analyzed by two-way ANOVA with Sidak’s multiple comparisons. Error bars represent means ±Std. Dev. ***p < 0.001; *p < 0.05

Beginning at 12 months of age, Tg4510 mice and littermate wild-type controls were fed chow containing PLX3397 (290 mg/kg, PLX3397) or control diet (Fig. 1a–c) for 3 months. By 15 months of age, Tg4510 mice on a control diet had an approximate 45% increase in the total number of Iba-1 positive microglia per cubic millimeter of cortical tissue compared to wild-type mice (wild-type 5975 ± 1060 microglia per mm^3^, Tg4510 8465 ± 1167 microglia per mm^3^; overall two-way ANOVA effect of genotype *p* < 0.001, treatment *p* < 0.001, interaction *p* = 7324; PLX-WT vs PLX-TG *p* = 0.012). Treatment of mice with PLX3397 resulted in a 30% reduction in the number of microglia in Tg4510 (5956 ± 1490 microglia per mm^3^; *p* = 0.007) and wild-type mice (3624 ± 1885 microglia per mm^3^; *p* = 0.02) compared to mice fed control diet. Notably, Tg4510 mice treated with PLX3397 had a density of microglia comparable to untreated wild-type mice (*p* > 0.999) though surprisingly, treating with this dose did not result in the > 96% elimination of microglia previously reported [10]. In total, we treated 16 mice with PLX3397 in this study and only observed 1 individual to have a > 96% depletion of microglia (Fig. 1b). Notably, this wild-type mouse was observed to have a greater body weight than cage mates and we postulate that it consumed a higher dose over the duration of the experiment. In general, our results are similar to those achieved by Dagher et al. in aged 3xTg mice treated with a related compound PLX5622 who also reported a 30% reduction in microglia between treated and untreated mice [8].

We hypothesized that one explanation for this discrepancy in effective dose could be related to changes in CSF1R expression, the target of PLX3397, with age and pathological tau accumulation. RNA was extracted and probed for CSF1R expression by quantitative PCR. We did not detect a change in relative expression levels of CSF1R between treated and untreated mice (Fig. 1d, two-way ANOVA *p* = 0.400), between genotypes (*p* = 0.408), or between 6- and 15-month-old mice (Fig. 1e, two-way ANOVA effect of age *p* = 0.273).

### Disease-associated microglial profiles in Tg4510 mice

Next, we examined gene expression from the brains of mice treated with PLX3397 to determine how the setting of inflammation is altered by treatment. Recently, others have identified “disease-associated” transcription profiles in mice that develop amyloid β plaques and we sought to include key markers of this microglial sub-type including *Apoe* and *Trem2*, in our analysis. Gene expression from total brain extracts indicate that expression levels of *Apoe*, *Cd68*, *Cx3cr1*, *Il1β*, *Tgfβ1*, and *Trem2* were all significantly upregulated in Tg4510 mice versus wild-type (Fig. 1f). *Cd68* (macrosialin) is a cell surface receptor that is upregulated in phagocytic microglia and in Alzheimer’s disease. In Tg4510 mice treated with PLX3397, *Cd68* was significantly upregulated compared to control diet. *Tgf1β* is a protein involved in maintaining microglial homeostasis and was also upregulated in Tg4510 mice fed PLX3397 versus control diet. Altogether, these data indicate that tau expression also upregulates a phenotype similar to that of amyloid induced pattern of “disease-associated” microglial gene expression and that reducing microglial numbers does not eliminate these gene changes.

### Tau pathology and phosphorylation is unaltered

**Fig. 2:**
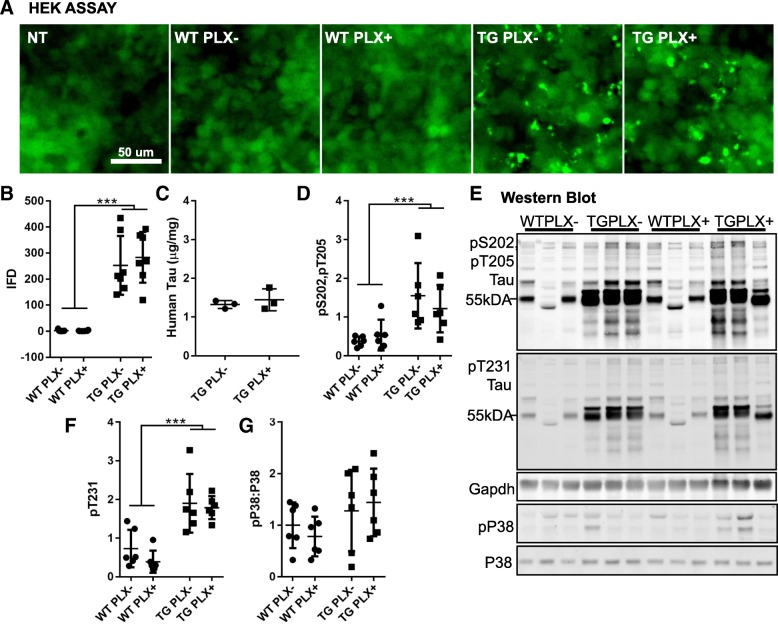
Tau characteristics in PLX treated and untreated mice are similar. **a** Images of HEK cells 16 h after treatment with 0.1 μg wild-type (WT) or Tg4510 (TG) brain homogenate. Non-treated (NT) cells were handled similarly with 1% lipofectamine in OPTIMEM but brain homogenate was omitted. Protein aggregates were easily seen with 488 nm fluorescent excitation in TG treated samples. **b** Integrated FRET density (mean fluorescence intensity x number of FRET+ cells) was quantified from an average of three wells per sample. **c** A total human tau ELISA from three randomly selected Tg4510 mice treated with or without PLX (Student’s *t* test, not significant). **d** Quantification of pS202, pT205 (AT8) labeling from the Western blot shown in (**e**) normalized to Gapdh. **e** Western blot of phosphorylated tau, Gapdh, active pP38, and total P38 in wild-type and Tg4510 mice. **f** Quantification of pT231 from panel (**e**) normalized to Gapdh. **g** Quantification of active pP38 ratio to total P38 from westerns shown in (**e**). Unless otherwise indicated, all data were analyzed by two-way ANOVA followed by Sidak’s multiple comparisons. Error bars represent means ±Std. Dev. ***p < 0.001

Having confirmed that we altered microglial density and gene expression via PLX3397 treatment, we next determined the effect of this manipulation on tau. Notably, microglia may play a role in spread of pathological tau from one brain region to another [1] as well as contribute to the post-translational modification of tau through kinase activity and phosphorylation [24, 29]. To explore this, we used a FRET-based HEK cell tau seeding assay to determine if reducing microglia also reduced the potential of tau to seed new aggregates through templated misfolding (Fig. 2a, b). Protein lysates from Tg4510 brain were capable of inducing FRET aggregates but not protein from wild-type brain as shown previously (two-way ANOVA, effect of genotype *p* < 0.0001). No difference was detected in the seeding activity of tau from PLX+ and PLX− mice (*p* = 0.6122). We confirmed that PLX treatment did not alter tau protein levels by randomly sampling Tg4510 mice from each group and measuring total tau by ELISA (Fig. 2c, Student’s *t* test *p* = 0.525).

We then looked at the phosphorylation state of tau in these mice, focusing on phosphorylation sites pS202/T205 and pT231 as these may be enhanced by active p38. While phosphorylation was increased in Tg4510 mice compared to wild-type, no difference was detected between PLX+ and PLX− mice (Fig. 2d–f). Similarly, when we assess active phospho-P38 by Western blot, no increase in the total ratio of pP38:total P38 was observed indicating that this does not play a significant role in this model at this age (Fig. 2e, g).

**Fig. 3:**
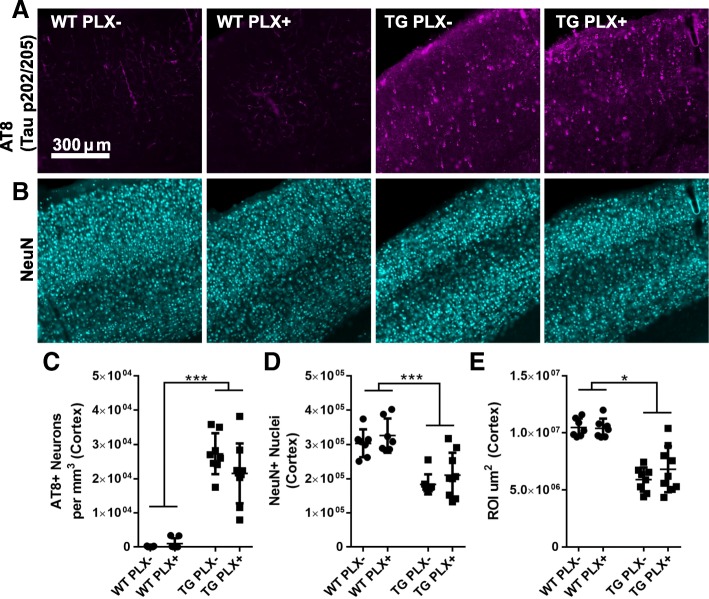
No change in location of tau deposition or neuronal loss. **a** Tau (pS202, pT205; AT8) labeling in somatosensory cortex of wild-type (WT) and Tg4510 (TG) mice. **b** NeuN labeling of neuronal nuclei in the same area of cortex. **c** AT8 quantification of the number of neuronal somas per cubic mm by stereology. **d** The total number of NeuN nuclei in cortex estimated by stereology. **e** The total region of interest (ROI) area per cubic micron measured for these analyses. All data were analyzed by two-way ANOVA. Error bars represent ±Std. Dev. ***p < 0.001; *p < 0.05

We further confirmed these biochemical observations of tau pathology by performing tau immunofluorescence in tissue sections using the antibody AT8 (pS202, T205; Fig. 3a, c). The total number of tau+ somas in the cortex was counted by stereology which confirmed no change in the number of AT8+ neurons in cortex (two-way ANOVA effect of genotype *p* < 0.001, TG PLX+ vs. PLX− *p* = 0.899).

### No change in neuron loss or count

Cortical atrophy is ongoing in Tg4510 mice at this age [4]. In accord with these observations, stereological counts of NeuN positive neurons in the cortex revealed neuronal number in the cortex was reduced by approximately 50% compared to wild-type mice by 15 months. To determine if reducing microglia may exacerbate or slow neurodegeneration process, we counted NeuN neurons in Tg4510 mice treated with PLX; no change was detected in the number of neurons observed in cortex in PLX+ versus PLX− Tg4510 mice (two-way ANOVA, effect of genotype *p* < 0.001).

### No change in blood vessel measures

**Fig. 4:**
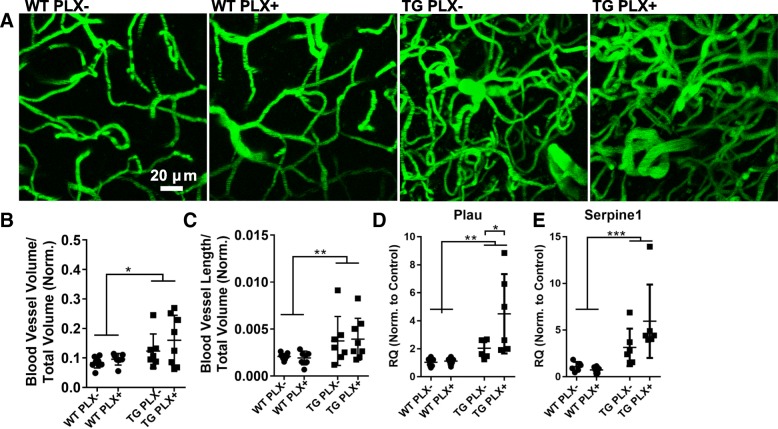
Blood vessel measures in PLX treated and untreated mice. **a** Representative z-stack (50 slices) of fluorescein-dextran labeled blood vessels viewed in vivo through cranial windows in wild-type (WT) and Tg4510 mice (TG). **b** The total blood vessel volume and **c** blood vessel length was measured from all images and is presented as a proportion of total imaged volume normalized to cortical thickness to account for atrophy. **d** Urokinase plasminogen activator (*Plau*) gene expression was measured by qPCR. **e** QPCR measurement of plasminogen activation inhibitor (*Serpine1*) gene expression. All data were analyzed by two-way ANOVA followed by Sidak’s multiple comparisons. Error bars represent means ±Std. Dev. ***p < 0.001; **p < 0.01; *p < 0.05

We have recently reported that blood vessel morphology is distorted in the cortex of aged Tg4510 mice with capillary density increasing between 12 and 15 months of age. To assess the possible contribution of microglia to this phenotype, we imaged blood vessels in mice via in vivo two-photon microscopy (Fig. 4a–c). Z-stack images acquired from somatosensory and motor cortex areas in PLX− and PLX+ Tg4510 mice appeared equally distorted with both groups showing similarly increased vascular density (Fig. 4b) and length (Fig. 4c) compared to wild-type mice. Further, in previous blood vessel studies, we identified gene expression changes associated with the morphological alterations. Two of the most highly upregulated genes, *Plau* and *Serpine1*, are also expressed by microglia. In these studies, we confirmed that *Plau* and *Serpine1* are both overexpressed in Tg4510 brain compared to wild-type (Fig. 4d, e) and attempted to determine the relative contribution of microglia to this increase. No reduction in the expression of either gene was observed, and surprisingly, *Plau* expression was increased in PLX+ versus PLX− mice indicating that reducing microglia may either have little impact on or may exacerbate these pathological blood vessel changes in Tg4510 mice.

### No change in GFAP-positive astrocytes

**Fig. 5:**
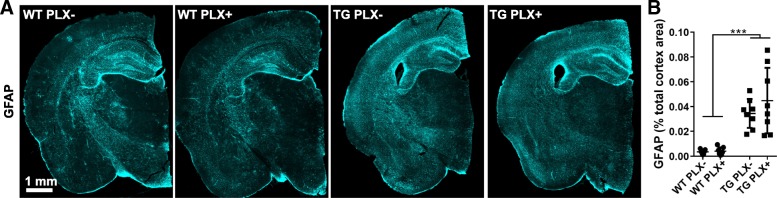
No change in astrocyte activation area. **a** Full slice images of GFAP-labeled area from wild-type (WT) and Tg4510 (TG) mice. **b** An intensity threshold-based measurement of the total GFAP area as a percentage of all measured area. Two-way ANOVA. Error bars represent mean ±Std. Dev. ***p < 0.001

Finally, to determine if PLX treatment or microglial decrease to control levels had an effect on astrocyte activation, we measured GFAP in these mice (Fig. 5a, b). Tg4510 mice had considerable elevation of GFAP particularly in the cortex (two-way ANOVA effect of genotype *p* < 0.001) and underlying white matter. Quantification of GFAP in cortex revealed no change between PLX+ and PLX− Tg4510 or control mice (*p* = 0.3261).

## Discussion

These studies indicate that reducing the number of microglia in the aged brain, after tangle formation and neuronal death has begun, may not prevent additional tau accumulation and related pathological changes. In these experiments, mice fed a diet containing PLX3397 were found to have a 30% reduction in the number of microglia in the cortex, so that the microglial complement of treated Tg4510 mice was numerically equivalent to that of controls. This intervention did not alter the extent of cortical neurodegeneration, assessed either by the number of neurons with AT8+ tau inclusions or the extent of neuronal loss, extent of astrocyte activation, or tau associated changes in vascular morphology. Additionally, this intervention did not modify p38-MAPK activation. This was a surprising finding given the large body of literature demonstrating the role of microgliosis in activation of neuronal p38-MAPK and associated tau phosphorylation, and a growing body of literature demonstrating crosstalk between microglia and astrocytes [25] and microglia and endothelial cells [26].

Importantly, we found that genes associated with “disease-associated” microglia near plaques such as *ApoE* and *Trem2* are also upregulated in the brains of Tg4510 mice [18]. This subset of microglia may thus not be unique to Aβ plaques. We also found that reducing the number of microglia in the brain by 30% does not result in a similar 30% reduction in the expression of these “disease-associated” microglial genes. One reason for this could include upregulation by other cell types including astrocytes in the case of *Apoe*, *Il1β*, and *Tgf1β.* However, *Trem2*, *Cx3cr1*, and *Cd68* are nearly exclusively expressed by microglia in the brain. Further, despite reduction of microglia in PLX-treated mice, *Cd68* and *Tgf1β* both were surprisingly significantly upregulated in PLX+ versus PLX− animals. This finding is not unique to the study: indeed, other studies utilizing PLX5622 to deplete microglia in the 3xTg AD mouse model have found that some inflammatory markers significantly increase despite reduced numbers of microglia, including tumor necrosis factor α (TNFα) and CXCL1 [8]. In other mouse models of neurodegeneration, depletion of microglia with PLX3397 also exacerbates inflammatory markers [7, 17, 42], suggesting this may be a common feature of microglia depletion during neurodegeneration—possibly indicating a compensatory upregulation of inflammatory factors in response to microglial depletion in the setting of ongoing neurodegeneration. Overall, these data suggest either that the microglia that remain after depletion may change as a consequence of anti-CSF1R treatment or that some of the tau-responsive, activated microglia become less sensitive to anti-CSF1R treatment. Furthermore, if the microglia remaining after depletion are indeed more reflective of a “disease-associated” phenotype, as our data and others’ suggest, this also may indicate that repopulation from these leftover microglia may not be beneficial for “resetting” since microglia are repopulated from the remaining microglia [15]. Further investigation into whether these repopulated microglia carry over similar inflammatory phenotypes, epigenetic signatures, or other cellular memory would be vital prior to therapeutic application of microglia repopulation.

Despite using a PLX3397 dose (290 mg/kg) reported to eliminate microglia from the brain, in these experiments, we did not achieve a similar level of reduction [10]. The number of microglia eliminated in aged Tg4510 mice was on the same order of reduction reported using a related drug PLX5622 at a similar dose (300 mg/kg) [8]. Here, it does not appear that the failure of the drug to completely eliminate microglia was due to up- or downregulation of CSF1R with aging or tau overexpression, although it is unknown if sensitivity to CSF1R blockade changes with age. Thus, considering that a single mouse in our experiment was observed to have a near complete lack of microglia, for complete elimination of microglia in future experiments a higher dose (1200 mg/kg) may be required as reported in Dagher et al. [8]. Another limitation of this study is that peripheral monocytes were not measured. CSF1R is also expressed in macrophage, neutrophils, and osteoclasts and it is unclear if these populations were similarly altered in these mice [6, 31]. Others have reported that splenic macrophage populations remain largely intact following PLX3397 treatment, though numbers are reduced at sites of inflammation [12, 20, 23, 39]. It is unclear how these peripheral cells may interact with tau pathology and neurodegeneration in this model, though these experiments indicate that PLX treatment at this dose is unlikely to have an impact.

These results are in line with other microglial manipulation models which have similarly observed little change in pathological tau deposition. Only when microglia numbers are reduced in acute models of tau spread, or in young mice, has depletion of microglia had an effect on abrogating tau pathological spread or phosphorylation [1]. However, virally expressed tau models do not allow evaluation of other phenotypes that may be microglial associated such as neuronal death, astrogliosis, blood vessel pathology, or chronic microgliosis. Additionally, depletion of microglia in younger mouse models of neurodegeneration appears to have a larger effect on mitigating AD pathology overall [1, 37] which may reflect an overall resistance of established pathology to treatment, age-associated differences in microglia activation [2], or a shifting phenotype of microglia in different stages of AD course [18]. Indeed, other investigators have shown that microglial inflammation pathways are robustly altered in Tg4510 mice and that these changes are detectible in young 4.7-month-old animals [41]. These age-associated and disease course-associated changes in microglia physiology indicate an important consideration for development of treatment strategies and suggest an important role for early intervention.

## Conclusions

In summary, while microglia are attractive targets in Alzheimer’s disease considering their contribution to the inflammatory response in disease, it is clear from these studies that a modest reduction in microglia is unlikely to change the outcome of late-stage tau pathology or reduce inflammatory gene expression. It is possible that a more severe depletion of microglia may lead to a change in neurodegeneration-related phenotypes, or that the effect of microglia is more marked on early phases of disease, prior even to the accumulation of tau aggregates in the brain. Thus, it is critical that we continue to investigate the specific factors responsible for these pathological alterations to understand the potential window of microglial intervention in both aging and disease.

## Abbreviations

Aβ: Amyloid β
CSF1R: Colony stimulating factor 1 R
FRET: Fluorescence resonance energy transfer
HEK: Human embryonic kidney
IFD: Integrated FRET density
PBS: Phosphate-buffered saline
PLX: PLX3397
TBS: Tris-buffered saline
TNFα: Tumor necrosis factor α
